# Connexin Mutants Compromise the Lens Circulation and Cause Cataracts through Biomineralization

**DOI:** 10.3390/ijms21165822

**Published:** 2020-08-13

**Authors:** Viviana M. Berthoud, Junyuan Gao, Peter J. Minogue, Oscar Jara, Richard T. Mathias, Eric C. Beyer

**Affiliations:** 1 Department of Pediatrics, University of Chicago, Chicago, IL 60637, USA; pminogue@peds.bsd.uchicago.edu (P.J.M.); ojaraleiva@peds.bsd.uchicago.edu (O.J.); ecbeyer@uchicago.edu (E.C.B.); 2 Department of Physiology and Biophysics, Stony Brook University, Stony Brook, NY 11794, USA; junyuan.gao@stonybrook.edu (J.G.); richard.mathias@stonybrook.edu (R.T.M.)

**Keywords:** cataract, intercellular communication, connexin mutant, calcium, calcification, biomineralization

## Abstract

Gap junction-mediated intercellular communication facilitates the circulation of ions, small molecules, and metabolites in the avascular eye lens. Mutants of the lens fiber cell gap junction proteins, connexin46 (Cx46) and connexin50 (Cx50), cause cataracts in people and in mice. Studies in mouse models have begun to elucidate the mechanisms by which these mutants lead to cataracts. The expression of the dominant mutants causes severe decreases in connexin levels, reducing the gap junctional communication between lens fiber cells and compromising the lens circulation. The impairment of the lens circulation results in several changes, including the accumulation of Ca^2+^ in central lens regions, leading to the formation of precipitates that stain with Alizarin red. The cataract morphology and the distribution of Alizarin red-stained material are similar, suggesting that the cataracts result from biomineralization within the organ. In this review, we suggest that this may be a general process for the formation of cataracts of different etiologies.

## 1. Introduction

Intercellular communication mediated by gap junctions is critical to the function of many organs. Gap junctions are plasma membrane specializations containing intercellular channels that allow the passage of cytoplasmic ions and small molecules (up to ~1 kDa) directly from cell to cell. They are formed by the coaxial alignment of two hemichannels; one is contributed by each of the closely apposed cells. The gap junction hemichannel is a hexameric assembly of protein subunits called connexins. Mutations of the genes encoding the connexins have been linked to a wide spectrum of diseases. The genetic disruption of the connexin-encoding genes can cause additional pathologies. Studies of the connexins expressed in the eye lens have provided unique insights into the mechanisms by which connexin mutants and loss of intercellular communication lead to disease. In this article, we summarize the findings of these studies and emphasize our recent observations.

## 2. The Lens and Its Specialized Cells 

**Figure 1:**
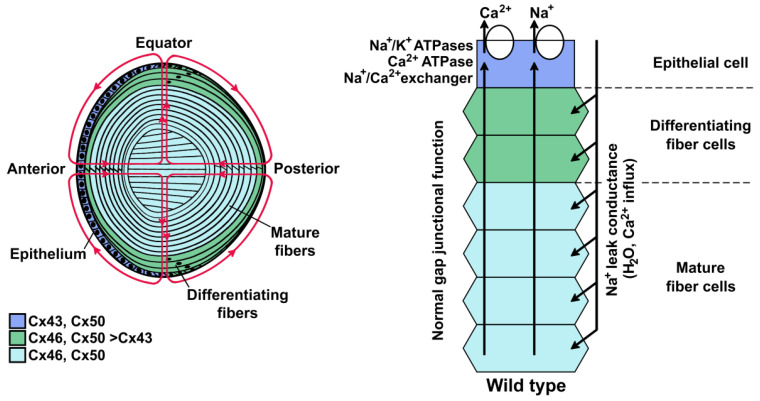
The lens and its internal circulation. Left, the diagram illustrates the distribution of connexins in the different regions of the lens and the circulation of ions and fluid (magenta lines with arrowheads to indicate direction of flow). Cells from the anterior epithelium express connexin43 (Cx43) and connexin50 (Cx50). Differentiating fiber cells express Cx43, connexin46 (Cx46), and Cx50, but the expression of Cx43 is downregulated as differentiation proceeds. Mature fiber cells express Cx46 and Cx50. In the lens circulation, ions and fluid enter into the extracellular spaces at the anterior and posterior poles and they exit across epithelial cell membranes at the equator. Right, the diagram depicts the ion circulation and some of the components that support it in a cellular column encompassing the lens surface to its center. Ions enter differentiating fiber and mature fiber cells through the plasma membrane driven by their electrochemical gradient, and they move back to the surface from cell-to-cell through gap junction-mediated electro-diffusion and advection. Once they reach the cells in the epithelium, the ions are transported out of the lens by pumps and transporters. Modified from Gao et al. (left panel, Figure 1; right panel, Figure 8A) [3].

The lens is an avascular organ formed by an anterior surface epithelium, with the bulk of the tissue formed by fiber cells. In the equatorial region, epithelial cells give rise to differentiating fiber cells, which become mature fiber cells through a process that involves cell elongation and loss of organelles, including the nuclei (Figure 1). The main function of the lens is to transmit and focus light onto the retina. This function is facilitated by several specializations. The loss of organelles minimizes light scattering. The fiber cells are narrow and organized with little extracellular space between them, minimizing light scattering at the cell borders. They contain a very high concentration of water-soluble proteins, referred to as crystallins, which have a liquid-like or glass-like, short-range spatial order that limits density fluctuations and light scattering [1,2].

## 3. Intercellular Communication and Healthy Survival of the Lens

Although fiber cells lose their organelles as they mature, various mechanisms ensure their survival. Fiber cells are connected to each other through specialized intercellular junctions, including gap junctions. In people, mature lens fiber cells express two connexins: connexin46 (Cx46) and connexin50 (Cx50).

The maintenance of cellular homeostasis in the lens is supported by an internal circulation system. This circulation is driven by the regional distribution and activities of ion channels, transporters, and exchangers (reviewed by Mathias et al. [4]). It allows ions and nutrients to reach the center of the lens and unwanted metabolites to move from the center to superficial cells, where they can be expelled. In the circulation model (Figure 1), ions (e.g., Na^+^ and Ca^2+^) enter the lens at the anterior and posterior poles and move to the center through the extracellular spaces. As the ions proceed towards the lens center, they are driven into fiber cells by their transmembrane electrochemical gradients. The outward movement of cytoplasmic ions from fiber cells in the lens center to the epithelial cells on the lens surface occurs by cell-to-cell diffusion, conduction, and advection through gap junction channels. The circuit of the lens circulation is completed when ions are transported out of the lens by epithelial Na^+^/K^+^-ATPases (for Na^+^) or by the epithelial Na^+^/Ca^2+^ exchanger and Ca^2+^-ATPase (for Ca^2+^). The ions do not move alone; their transmembrane fluxes create transmembrane osmotic gradients, coupling the movement of fluid to the circulation of ions (Figure 1). Under normal conditions, the lens may compensate for a brief disturbance of the lens circulation by increasing/decreasing the activities of ion channels, solute carriers, transporters, and/or exchangers.

## 4. Lens Disease and Connexins

The most common disease of the lens is the development of cataracts. A cataract is a cloudiness or opacity in the lens that disrupts normal light transmission and its focusing onto the retina. Cataracts are the leading cause of blindness worldwide [5]. They are often associated with aging and can be caused by a variety of diseases and environmental factors. Congenital cataracts are the most common cause of childhood visual impairment, accounting for 10% of childhood blindness [6]. About half of congenital cataract cases have a genetic basis, with many linked to mutations in the genes encoding major lens proteins (including those of Cx46 and Cx50).

Cataract-associated mutations have been identified at more than 40 different positions in the genes encoding Cx46 (*Gja3*) and Cx50 (*Gja8*) in human pedigrees [7]. Most of the connexin mutants lead to cataracts with an autosomal dominant inheritance pattern, and most of the mutants that have been investigated are non-functional when studied using expression systems (reviewed in Berthoud and Ngezahayo [8]). Since gap junctions are an important component of the lens circulation, the results of the in vitro studies suggest that most cataract-linked lens connexin mutants will reduce lens gap junctional coupling and affect the lens circulation. In addition, connexin mutants with increased hemichannel activity (but normal gap junction channel activity) could potentially affect the lens circulation by depolarizing the cells, which would decrease the driving force for the movement of ions throughout the organ.

Because it is not possible to study the consequences of Cx46 or Cx50 mutants expressed in human lenses, these mutants have been assessed in mouse models. Mice with homozygous (but not heterozygous) targeted deletion of Cx46 or Cx50 develop cataracts [9,10,11]. Mice with mutations in the Cx46 or Cx50 genes develop cataracts that are inherited with a dominant or semi-dominant pattern [12,13,14,15,16,17]. The lenses of mice with targeted deletion of Cx46 or Cx50, or with dominant mutations in either of these genes, have very reduced levels of Cx46 and/or Cx50 [10,11,16,17,18] and of intercellular communication between lens fiber cells (measured as an increase in intracellular resistivity) [18,19,20,21]. Transgenic mice with lens over-expression of a mutant ubiquitin (K6W-Ub) that is conjugation competent but proteolytically incompetent also have decreased gap junctional coupling between fiber cells in the core of the lens, likely because these mice have lower levels of Cx46, as detected by immunoblotting [22]. These results suggest that any gene mutation/deletion or insult that leads to a drastic decrease in the abundance and/or function of connexins in lens fiber gap junctions may reduce coupling between fiber cells.

Other parameters related to the lens circulation have been determined in these lenses. The hydrostatic pressure is increased in lenses from heterozygous Cx46 knockout mice and lenses from mice expressing Cx46 or Cx50 dominant mutants [3,21,23]. The intracellular concentration of Na^+^ is increased in lenses from Cx46 mutant mice [3]. The intracellular concentration of Ca^2+^ ([Ca^2+^]_i_) is increased in the lens fiber cells from homozygous Cx46 knockout mice, transgenic K6W-Ub mice, and mice expressing Cx46 or Cx50 mutants [3,21,22,24]. The emerging picture from these results is that decreased intercellular communication between lens fiber cells leads to an increased gradient of hydrostatic pressure and elevated gradients of the intracellular concentrations of sodium ions and calcium ions [3,21,22,23,24]. It is likely that gap junctional coupling is also impaired in cataractous lenses from mice homozygous for the γB-crystallin S11R mutation, because these lenses have severely decreased levels of Cx46 and Cx50 and increased total Ca^2+^. This inference is supported by the observation that the knock-in of Cx46 into the Cx50 locus suppressed the nuclear cataract [25]. Extrapolation to humans suggests that people carrying a mutation or suffering a lens insult that severely decreases connexin levels or gap junction function likely develop cataracts due to impairment of the lens circulation.

## 5. Calcium Ions in the Normal and Cataractous Lens

As in other cell types, Ca^2+^ homeostasis in the lens fiber cells depends on the binding/sequestration of intracellular Ca^2+^ and on the balance between ion influx and efflux. The [Ca^2+^]_i_ in the lens can be modulated through storage of the ion in organelles, including the endoplasmic reticulum and mitochondria, and through its binding by cytoplasmic proteins. The organellar sequestration of Ca^2+^ can only occur in surface cells, because the organelles are absent in the mature fiber cells. Calcium-binding cytoplasmic proteins (including calmodulin and some crystallins) contribute to the buffering of Ca^2+^ in all cells, and they provide the only mechanism in mature fiber cells.

Lens Ca^2+^ influx depends on entry through plasma membrane channels in interior fiber and surface cells. Voltage-dependent Ca^2+^-selective channels have been identified in lens epithelial and surface fiber cells, as have other channels that would allow Ca^2+^ permeation, such as TRP channels [26,27,28,29]. The movement of extracellular Ca^2+^ from the surface of the lens to its center is driven by diffusion, conduction, and advection. As extracellular calcium ions move inward, they are driven by the Ca^2+^ transmembrane electrochemical gradient to enter the more central cells through the fiber cell membrane’s Ca^2+^ permeability. Lens intracellular calcium ions move through gap junctions to surface cells where efflux is driven by active transport by the Ca^2+^-ATPase and Na^+^/Ca^2+^ exchanger; it may be influenced by a recently identified K^+^-dependent Na^+^/Ca^2+^ exchanger (Nckx4) in outer cortical fibers [30].

How is the Ca^2+^ efflux from surface cells connected to the Ca^2+^ influx in mature fiber cells located in more central regions of the lens? These spatially distant processes can be coordinated because the extensive network of lens fiber gap junction channels provides coupling throughout the organ. Gap junction-mediated intercellular communication allows the electro-diffusion and advection of Ca^2+^ from the center of the lens towards the site of active ion transport in surface cells.

**Figure 2:**
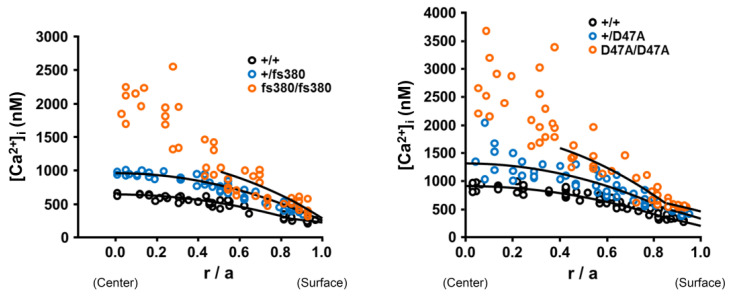
Intracellular calcium ion concentrations are increased in Cx46fs380 and Cx50D47A lenses. Graphs show the intracellular calcium concentrations ([Ca^2+^]_i_) in wild type (black circles), heterozygous (blue circles) and homozygous (orange circles) Cx46fs380 (left) and Cx50D47A (right) lenses as a function of radial distance from the lens center (r; cm), normalized to the lens radius (a; cm). The curves represent the best fit to the data based on the model described in Gao et al. [24]. Reproduced from Gao et al. (Figure 6J) [3] and Berthoud et al. (Figure 6D) [21].

In the normal (wild type) mouse lens, the [Ca^2+^]_i_ gradually increases from the lens surface to the center, forming a gradient that can be described by a parabolic function [24]. However, in the lenses of connexin mutant mice, Cx46-null mice, or mice with substantially decreased Cx46 levels, the [Ca^2+^]_i_ increases beyond the levels in wild type lenses at all radial distances from the lens center [3,21,22,24]. The extent to which the radial [Ca^2+^]_i_ can be fit to a parabola differs in the mutant mouse lenses. In the Cx46 and Cx50 mutant mice, it fits only up to a partial depth into the lens (from the lens surface); the [Ca^2+^]_i_ in the central region does not fit to this equation (Figure 2) [3,21]. Thus, in the centers of the connexin mutant lenses, the influx of Ca^2+^ surpasses efflux and buffering, and calcium ions accumulate. Mutant lens connexins with increased hemichannel activity may also lead to significant increases in the [Ca^2+^]_i_, as these hemichannels provide a conduit for the equilibration of the intracellular and extracellular concentrations of all permeant ions and molecules, including Ca^2+^.

## 6. The Cataractous Lens: A Mineralized Organ?

The biochemical changes present in lenses with cataracts due to different causes have been extensively studied. Abnormalities include the formation of high molecular weight protein aggregates, post-translational modification of lens proteins, protein cleavage, and protein degradation (reviewed in [31,32,33]). Calcium has also been implicated in cataract pathogenesis [34]. Human and mouse lenses with cataracts of different etiologies contain increased Ca^2+^ [22,24,25,35,36,37]. As noted above, we found an increased [Ca^2+^]_i_ in the lenses of mice carrying a Cx46 or Cx50 mutant [3,21].

Because calcium salts have relatively low solubilities in aqueous solutions and the [Ca^2+^]_i_ detected in cataractous lenses [3,21,22,24] reaches values of >1000 nM in the center of the lens, it seemed likely that Ca^2+^ would precipitate. This hypothesis is supported by reports of increased calcium associated with the insoluble lens fraction (as compared with the soluble fraction) of cataractous human lenses [35] and the finding of calcium oxalate or calcium carbonate crystals in human cataractous lenses [38,39,40].

**Figure 3:**
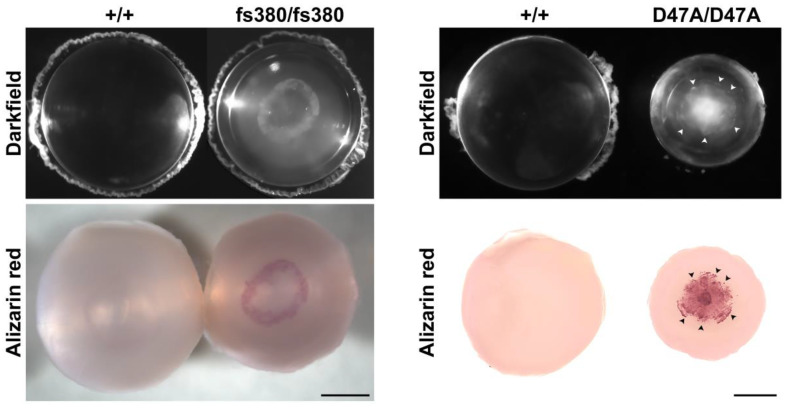
Alizarin red stains Ca^2+^-containing precipitates in Cx46fs380 and Cx50D47A cataracts. Lenses from wild type (+/+) and homozygous Cx46fs380 (fs380/fs380) or Cx50D47A (D47A/D47A) mice were photographed using darkfield illumination (top panels) and after whole-mount staining with Alizarin red (bottom panels). Arrowheads in the homozygous Cx50D47A lens point to the Alizarin red-stained precipitates that can be mapped to the cataract outline. The agreement between the Alizarin red staining and the cataract shape suggests that the spatial distribution of the Ca^2+^-containing precipitates determines the cataract morphology. Scale bars: 750 and 780 µm for darkfield and Alizarin red-stained images of Cx46fs380 lenses, respectively; 591 µm for Cx50D47A lenses. Modified from Gao et al. (Figure 7, Cx46fs380 panels) [3] and Berthoud et al. (Figure 7, Cx50D47A panels) [21].

We identified immobile Ca^2+^ in cataractous lenses from mice expressing Cx46 or Cx50 mutants by whole-mount staining with Alizarin red, a dye used to detect insoluble Ca^2+^ in other tissues (Figure 3). Interestingly, performing the staining in whole-mounts allowed us to observe that the pattern of Alizarin red staining resembles the morphology of the cataract [3,21]. This observation suggests that these cataracts are largely generated by the formation of insoluble calcium salts (minerals).

## 7. Cataracts May Result from Biomineralization in the Lens

**Figure 4:**
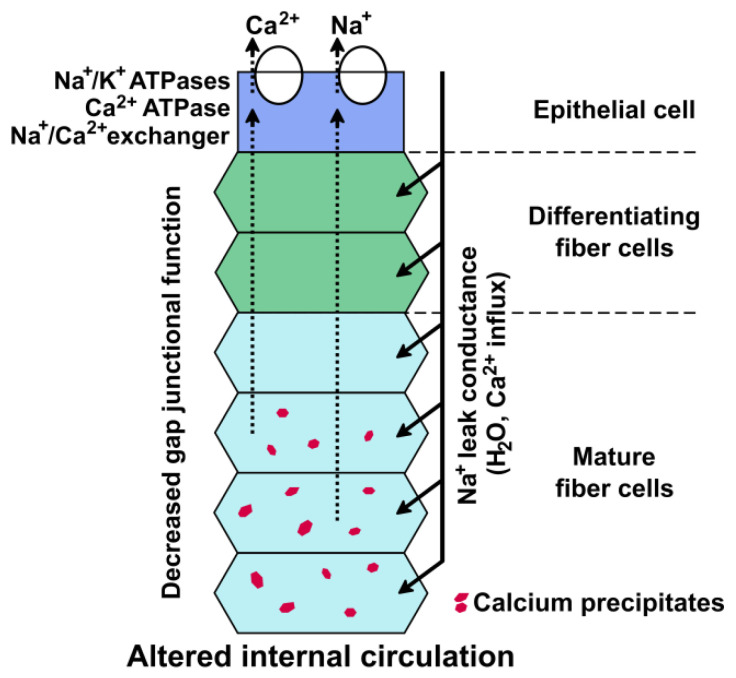
Lens connexin mutants alter the lens circulation. The diagram shows the lens circulation in a cellular column of a homozygous connexin mutant lens to illustrate the effects of the expression of the connexin mutant. The expression of a mutant connexin decreases the gap junction-mediated circulation of ions (dotted lines). An increase in [Na^+^]_i_ leads to a decrease in the activity of the Na^+^/Ca^2+^ exchanger. An increase in [Ca^2+^]_i_ above 1 μM exceeds the buffering capacity, resulting in the formation of Ca^2+^-containing precipitates. Modified from Gao et al. (Figure 8B) [3].

Biomineralization is the formation of mixed deposits comprised of organic materials and insoluble precipitates/crystals containing inorganic ions [41]. It occurs normally in some tissues (such as bone and teeth), but it is a pathologic process in others (such as the generation of kidney stones and calcification of blood vessels) [42]. The initial step in biomineralization is the formation of a nucleation particle. Subsequently, other material deposits upon this particle. In lenses with an impaired lens circulation that causes Ca^2+^ accumulation (as in mice with Cx46 or Cx50 gene mutations), we postulate that the first step is the precipitation of calcium salts after the intracellular concentrations of Ca^2+^ and the corresponding anions surpass their *K*_sp_ (Figure 4).

**Figure 5:**
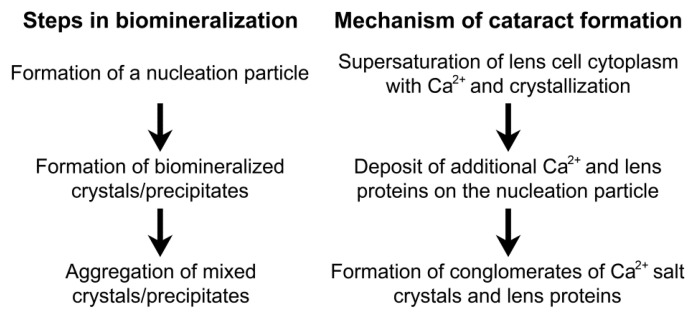
The proposed mechanism by which calcium ions contribute to cataract formation through biomineralization. Lenses from mice with disruptions of the lens circulation due to mutations of Cx46 or Cx50 accumulate Ca^2+^. When the *K*_sp_ for calcium salts is surpassed, the salts precipitate and act as a nucleation particle. Then, more calcium salts and lens proteins deposit over the nucleation particle. With time, this leads to the formation of an aggregate containing a mix of calcium salts and lens proteins, i.e., a deposit of biomineral.

Although biomineralization of bone and teeth occurs in the extracellular matrix, it is likely that the initial formation of precipitated calcium salts occurs inside the lens fiber cells of the connexin mutant lenses, where the intracellular concentrations of calcium ions and their counterions have surpassed their *K*_sp_. These precipitates act as nucleation particles for the deposit of other molecules, including proteins, lipids, and/or additional Ca^2+^ (or other cation) salts (Figure 5). As the calcium precipitates grow, they can remain inside the cell or they may eventually disrupt the integrity of the plasma membrane and become extracellular. These biomineral deposits are detected as cataracts.

Alterations of the lens circulation and subsequent biomineralization in the lens may be affected by modifications of the connexins. The lens fiber connexins can undergo several post-translational modifications, including acetylation, phosphorylation, ubiquitination, carbonylation, nitrosation, and cleavage [43,44,45,46,47,48,49]. Their effect on gap junction or hemichannel function is known in some cases. The modeling of gap junction intercellular channels made of Cx46 and/or Cx50 suggests that N-terminal acetylation of these connexins increases their cation-to-anion specificity [47]. The effects of phosphorylation depend on the protein kinase, the substrate amino acid residue, and the connexin subtype. It can alter gap junction intercellular communication and channel and hemichannel permeability [50,51,52,53,54,55], and can also affect connexin stability [56,57]. Cleavage has been implicated in changing the pH sensitivity of the lens fiber connexin channels, but this effect varies between connexin subtypes and animal species [58,59,60,61,62]. Cleavage can be regulated by phosphorylation [63]. The S-nitrosation of Cx46 (induced with NO donors) increases its hemichannel tail currents and inactivation at positive voltages of 50–60 mV, but does not affect the electrophysiological properties of the Cx46 gap junction channels [48]. The carbonylation of Cx46 (induced with 4-hydroxynonenal) decreases its hemichannel currents and dye uptake [49].

Our model for cataract formation may also have implications regarding the pathogenesis of age-related cataracts. Since lens fiber cell connexins have a much longer half-life than other members of the connexin family and mature lens fiber cells lose the synthetic machinery necesssary to replace old/damaged connexins by newly synthesized ones, the connexins can accumulate modifications with aging. These modifications may cause the deterioration of gap junctional intercellular communication and/or an increase in connexin hemichannel activity (and thereby an impairment of the lens circulation), leading to an increase in the [Ca^2+^]_i_ to a sufficiently high level that calcium ions start precipitating. However, the healthy aging lens must also have protective/compensatory mechanisms, since the lenses of 14-month old mice can remain free of cataracts, despite reduced lens intercellular communication and Ca^2+^ accumulation [64].

## 8. Potential Generality of the Biomineralization Model for Cataract Formation

Our results suggest that any process that impairs the lens circulation (such as mutations in the lens fiber cell connexin genes) can cause cataracts through Ca^2+^ accumulation, precipitation, and biomineralization. The insoluble material in these cataractous lenses likely contains aggregated or denatured proteins, in addition to the precipitated Ca^2+^ salts. Lens biomineralization might be the key to the formation of cataracts of other etiologies. In other systems/tissues, various proteins participate in initiating the process of mineralization [65]. Thus, in cataracts that do not start through disruption of the lens circulation, the initial step might be the denaturation and/or aggregation of lens proteins that subsequently act as a surface for the deposit of Ca^2+^ salts, followed by the growth of mixed aggregates detectable as significant opacities.

## Abbreviations

[Ca^2+^]_i_	Intracellular concentration of calcium ions
Cx43	Connexin43
Cx46	Connexin46
Cx50	Connexin50
K6W-Ub	Ubiquitin K6W mutant
*K*_sp_	Solubility product constant
NO	Nitric oxide
